# Validation of a previous day recall for measuring the location and purpose of active and sedentary behaviors compared to direct observation

**DOI:** 10.1186/1479-5868-11-12

**Published:** 2014-02-03

**Authors:** Sarah Kozey Keadle, Kate Lyden, Amanda Hickey, Evan L Ray, Jay H Fowke, Patty S Freedson, Charles E Matthews

**Affiliations:** 1 Nutritional Epidemiology Branch, Division of Cancer Epidemiology and Genetics, National Cancer Institute, Bethesda, MD 20892, USA; 2 Cancer Prevention Fellowship Program, Division of Cancer Prevention, National Cancer Institute, Bethesda, MD, USA; 3 Division of Endocrinology, Metabolism and Diabetes, University of Colorado Denver Aurora, Aurora, CO 80045, USA; 4 Department of Kinesiology, University of Massachusetts, Amherst, MA 01003, USA; 5 Department of Math and Statistics, University of Massachusetts, Amherst, MA 01003, USA; 6 Division of Epidemiology, Department of Medicine, Vanderbilt University Medical Center, Nashville, TN 37232, USA

**Keywords:** Exposure measurement, Physical activity, Behavioral epidemiology

## Abstract

**Purpose:**

Gathering contextual information (i.e., location and purpose) about active and sedentary behaviors is an advantage of self-report tools such as previous day recalls (PDR). However, the validity of PDR’s for measuring context has not been empirically tested. The purpose of this paper was to compare PDR estimates of location and purpose to direct observation (DO).

**Methods:**

Fifteen adult (18–75 y) and 15 adolescent (12–17 y) participants were directly observed during at least one segment of the day (i.e., morning, afternoon or evening). Participants completed their normal daily routine while trained observers recorded the location (i.e., home, community, work/school), purpose (e.g., leisure, transportation) and whether the behavior was sedentary or active. The day following the observation, participants completed an unannounced PDR. Estimates of time in each context were compared between PDR and DO. Intra-class correlations (ICC), percent agreement and Kappa statistics were calculated.

**Results:**

For adults, percent agreement was 85% or greater for each location and ICC values ranged from 0.71 to 0.96. The PDR-reported purpose of adults’ behaviors were highly correlated with DO for household activities and work (ICCs of 0.84 and 0.88, respectively). Transportation was not significantly correlated with DO (ICC = -0.08). For adolescents, reported classification of activity location was 80.8% or greater. The ICCs for purpose of adolescents’ behaviors ranged from 0.46 to 0.78. Participants were most accurate in classifying the location and purpose of the behaviors in which they spent the most time.

**Conclusions:**

This study suggests that adults and adolescents can accurately report where and why they spend time in behaviors using a PDR. This information on behavioral context is essential for translating the evidence for specific behavior-disease associations to health interventions and public policy.

## Background

Currently, a modest percentage of Americans are meeting physical activity guidelines [1] and the majority of one’s time is spent in sedentary behaviors [2]. There is strong evidence that an insufficient amount of moderate-to-vigorous physical activity (MVPA) is associated with increased risk of mortality, cardiovascular disease, type 2 diabetes and some cancers [3]. There is increasing evidence that high levels of sedentary behavior are associated with negative health outcomes [4,5]. Therefore, interventions are needed to improve physical activity levels and decrease sedentary behavior [6]. We spend time each day in many different behaviors and our choices reflect our actions and inactions in response to both internal and external cues [7]. Accordingly, theories of health behavior, such as the socio-ecological model [8,9] and dual-process theory [10] consider the reciprocal relationships between the individual and their physical and social environment(s) as important determinants of behavior. Here we operationally define behavioral context as a combination of the location (where) and purpose (why) behavior is taking place. In order to design effective interventions and inform public policy, measurement tools that quantify specific physically active and sedentary behaviors within the relevant behavioral contexts are needed.

Current technologies can provide an objective assessment of the context of active and sedentary behaviors (e.g. Geographic information systems (GIS) or the SenseCam) but are relatively costly and require time-intensive data coding and processing [11,12]. Ecological momentary assessment methods have also been employed to gather contextual information about physical activity [13,14] but this method is less efficient for gathering estimates of total amounts of active or sedentary time over the entire day. Short-term recalls may be a feasible and valuable method to gather contextual information about active and sedentary behaviors [15-19]. We recently demonstrated the validity of a Previous Day Recall (PDR) in estimating active and sedentary time [15]. However, while our report suggests that the PDR provides accurate estimates of total active and sedentary time (hrs/d) compared to the activPAL [15], the accuracy of the PDR for classifying the context in which the activities occurred (i.e., location and purpose) has not been studied. Such details allow for the mapping of human activities to specific behavioral settings relevant to social ecological models of health behavior [8,9]. PDRs may be particularly valuable in studies understanding where and why activity-related behaviors occur. The objective of this report is to examine the validity of the PDR for classifying the location (e.g., community, school/work) and purpose (e.g. home activity, transportation) of behavior compared to a criterion of direct observation. In addition, the accuracy of the PDR for classifying time spent in active and sedentary behaviors within each context was examined.

## Methods

The study population and recruitment processes have been previously described [15]. Briefly, the study population included a sub-sample of participants that provided reliable recalls in an earlier investigation of physical activity/sedentary behavior assessment methods development (n = 219) in adolescents (12–17 yrs, N = 117) and adults (18–71 yrs, N = 102) recruited by the University of Massachusetts (Amherst, MA) and Vanderbilt University Medical Center (Nashville, TN). A comparison of the sub-sample to the overall study sample is in Table 1. In a seven day period, study participants completed three unannounced PDRs and wore ActiGraph and activPAL activity monitors [15]. Participants enrolled at the Amherst study site (n = 91) were offered the opportunity to participate in the direct observation sub-study, and 15 adolescents and 15 adults consented to the study. Adult participants and parents of the adolescents completed an informed consent document and adolescents provided assent. All study activities and documents were approved by the Institutional Review Boards at the University of Massachusetts. These participants agreed to be directly observed by a trained research assistant as they went about their normal daily activities. An unannounced PDR was completed on the day following observation.

**Table 1:** Descriptive characteristics of population and segment durations

	**Adults**		**Adolescents**	
	**Total sample**	**DO subset**	**Total sample**	**DO subset**
**N**	102	15	117	15
**Age (yrs)**	41.3 (14.8)	33.1 (11.5)*	14.3 (1.7)	14.5 (1.8)
**BMI**	26.9 (5.4)	26.6 (6.5)	21.4 (4.5)	20.4 (2.6)
**Female N (%)**	51 (53.7%)	8 (53.3%)	53 (49.1%)	6 (44.4%)
**% Sedentary**	62.6 (11.9)	58.4 (12.6)	68.3 (10.4)	66.3 (13.5)
	**DO**	**PDR**	**DO**	**PDR**
**Morning (min)**	274.1 (60.9)	298.0 (66.1)	202.5 (42.9)	226.7 (64.5)
**Afternoon (min)**	266.7 (101.6)	316.6 (108.9)	290.6 (49.2)	303.9 (54.0)
**Evening (min)**	140.0 (45.6)	151.9 (44.5)	177.3 (46.6)	211.8 (45.9)

Note: Total sample refers to Matthews et al. [15], from which the direct observation (DO) subset was recruited. PDR is previous day recall. Values are mean (standard deviation) unless otherwise indicated. *Indicates significant difference between overall sample and DO sample (P < 0.05).

### Direct observation protocol

Observers completed both didactic and experiential training. The didactic portion was administered by the direct observation study leader (KL) and was comprised of a face-to-face session that included a general orientation to the direct observation method as well as technical orientation to the electronic observation system. The experiential portion consisted of face-to-face practice training using a training video as well as 10 or more hours practice observing individuals in a free-living environment. Before data collection was initiated all observers had to demonstrate acceptable accuracy (>90%) in coding active and sedentary behaviors in the video, and the location and purpose of the behaviors in the text examples. A similar direct observation system from our group was recently shown to be highly accurate and precise compared to indirect calorimetry [20].

Participants were directly observed during at least one segment of the day (i.e., morning, afternoon or evening) and up to all three segments depending on participant availability. These segments were consistent with the PDR segments of the day. The morning segment was the time from arising from bed for the day until lunchtime (or 12:00 PM if no lunch). For the morning segment, observers arrived 30 minutes after the participant got out of bed to allow privacy to get dressed/undressed. The afternoon segment was the period during lunch (or 12:00 PM) until dinner (or 6:00 PM if no dinner). Observers arrived 30 minutes before the anticipated lunch time, began observing once they began lunch and continued until the participant ate dinner. The evening segment was the time after dinner until getting into bed for the night. Observers arrived 30 minutes before the anticipated dinner time and started observing once the participant was eating dinner. They were observed until 30 minutes before they planned to go to bed or until 10:00 PM.

During the observation period, participants were instructed to complete their normal daily routine and avoid interacting with the observer. The observer was instructed not to initiate conversation with the participant, to maintain a comfortable distance and to mimic the participant’s behavior when possible (i.e., sitting when the participant was sitting). The observer used a hand-held personal digital assistant programmed with custom software (Noldus Inc., Wageningen, Netherlands) to record participant behavior. Direct observation was conducted using continuous focal sampling where each activity change is coded, regardless of the duration between activities. For this study, every time behavior changed, the observer recorded the location, purpose and body-position/intensity of the activity. To minimize the amount of coding required, the purpose and body-position/intensity were recoded every time the activity/intensity changed, but the location was only recoded when it changed. “Private Time” was coded if a participant requested private time (e.g. to use the bathroom or change clothes). These instances were deleted when the data were processed. The coding scheme is described below and corresponds to the same classification approach used in the PDR.

*Location* is the physical domain where a behavior takes place

1. **Home:** Activities done on “household” property, either inside or outside the house. Activities were coded as indoor or outdoor, but were combined for analysis.

2. **Work/School:** Activities done at the work site (for pay), or on school grounds.

3. **Community:** Activities done away from home, but not at work or school locations.

*Purpose* of the activity is meant to describe why the participant is doing the activity. The category definitions are adapted from those of the American Time Use Survey [21] and the major headings from the Compendium of Physical Activities [22]. Some categories were merged to assist observers in coding and ensure sufficient sample size within each purpose. The same behavior/activity type could be coded with a different purpose, depending on the intention. For example, using a computer to shop would be considered leisure time, while using a computer to write a manuscript would be considered work. In addition, the purpose does not designate the location of the activity. For example, “office work” can take place in the community (working on a computer in the library), at home (sitting at computer) or at work (filing papers).

1. **Household activities:** This category is the combination of the following three purposes. They were combined after data collection due to low numbers.

a. **Household chores:** These activities are done by a person to maintain a household or care for another individual (i.e., elder or child). Examples include chores, meal preparation, child-care.

a. **Self-Care:** Includes grooming (bathing or dressing), health-related self-care, eating and drinking [21].

a. **Lawn and garden:** Activities that were related to maintaining outdoor space. Examples include gardening, raking, trimming, farming, or mowing the lawn.

2. **Work:** Includes time spent working, engaging in income-generating activities (outside ones job) and looking for work [21]. The observers could code this as the following two options.

a) **Office work:** This is work completed in an indoor setting. Examples include filing papers, working at computer (not including computer games).

a) **Physical work:** This category included activities that were for the purpose of work that involved manual labor. Examples include painting, construction, plumbing, electrical work, or fixing floor.

3. **Education:** This category includes activities related to school or university including research, homework and taking classes. This was only an option if the participant was a student.

4. **Transportation:** This category includes activities for the purpose of getting from one place to the next. For example, riding a bus, walking, bicycling, driving or riding in a car.

5. **Leisure:** Includes sports, recreation, socializing, personal interest activities (e.g., going to a museum or concert), playing games, watching television, listening to music and other leisure activities.

6. **Miscellaneous:** This category is for activities for which the observer was unclear of the purpose. They were instructed to make a note describing the activity for it to be recoded by PI (if appropriate) or left as miscellaneous.

*Body position and intensity level* were collected using the protocol from Kozey-Keadle et al. [23] and combined into the broader categories below.

1. **Sedentary behaviors:** Any behavior that was done while sitting, reclining, or lying down and that did not require substantial energy expenditure (typically < 1.5 metabolic equivalents [METS]) [24].

2. **Physically active behaviors:** Activities preformed while upright including standing still and moving. Exercise, sports and active recreation pursuits were classified as active regardless of body position (i.e., biking).

### Previous Day recall

The recall employed was an updated version of an earlier recall instrument [25,26], now called the PDR. In addition to physical activity, the instrument now gathers more detailed information about sedentary behaviors and explicitly classifies behaviors by their location and purpose. A detailed description of the PDR is provided in a previous paper [15]. Interviewers were certified to complete the PDRs using a standard training protocol composed of didactic and experiential training sessions designed to develop interviewing skills, expertise in interacting with the computer interface, and the integration of these two skills. During the study, interviewers led participants chronologically through the previous day (midnight to midnight) using a semi-structured interview based on methods developed and refined for the 7-Day Physical Activity Recall [27]. Interviewers gathered information about specific active and sedentary behaviors reported in three segments of the recall day (i.e., morning, afternoon, evening). Individual behaviors lasting at least five minutes in a given time-period were recorded/coded and the activity duration of the activities was entered directly into a database. Each behavior reported was coded as physically active or sedentary using reported body position and activity type (i.e., all exercise and sports pursuits were classified as “active”). The location and purpose of behaviors reported was classified as described above. Each activity in the database was derived from the Compendium of Physical Activities, along with the associated MET values [22]. To summarize the recall data, we summed the duration estimates of the behaviors for overall time reported by location and purpose and for both sedentary and active behaviors.

### Data synchronization

The PDR data for specific behaviors were not time stamped, so the times reported at the beginning and end of each segment of the day were used to synchronize the PDR and direct observation data for each segment of observation. The PDRs were completed on the day after the direct observation day and, after the recall was complete, the interviewers asked participants about activities they did when the observer was not present (i.e., before observer arrived or after they left) within the targeted segment. The activities the participant reported completing while the observer was not present were manually removed from the file and verified by a second researcher (SKK, AH or CM). For example, if the participant reported showering for 20 minutes before the observer arrived then 20 minutes of self-care was removed from the PDR summary file so that the length of the observation was the same as the PDR. There were two instances that could not be adjudicated (difference between PDR and direct observation was > 1 hour) because the PDR was unreliable. These PDR estimates remained in the analytic dataset.

### Statistical evaluation

Differences in age, BMI and sedentary time between the direct observation sample and the overall study sample were tested using a t-test with P < 0.05 indicating significant differences. For each location and purpose, PDR estimates were compared to direct observation for the total time (regardless of intensity) and then separately for active and sedentary behaviors. To do this, three statistical approaches were used. First, bias was estimated by subtracting PDR from direct observation values using repeated measures mixed effects models to account for multiple segments/observations for some individuals. To assess statistical significance of the bias 95% confidence intervals (CI) were used. Second, classification accuracy between measures was compared using percent agreement and the Kappa statistic with McNemar’s Test for statistical significance. The following interpretations are generally assigned to Kappa values (0 is chance agreement; 0.01-0.20 is slight agreement, 0.21-0.4 is fair agreement; 0.41-0.60 is moderate, 0.61-0.80 is substantial and 0.81-1.0 is strong agreement) [28]. Third, we used ICC to evaluate the level of agreement between the duration estimates using a two-way analysis of variance model. For all analyses, the N was defined as the number of segments where a participant spent at least one minute in a particular location/purpose according to direct observation. For example, if 12/26 participants spent time in the community, bias estimates for community only included those 12 observations. We used this more conservative approach because some activities were only completed by a few participants (Table 2), and because including all individuals, even if they did not complete the activity as per direct observation would make the bias artificially small (bias would be 0 since both direct observation and PDR would be 0). Similarly, mean values of time spent in each location/purpose (Table 2) are only for those participants who spent at least one minute in that location/purpose according to direct observation.

**Table 2:** Comparison of time spent in activity location and purpose using previous day recall and direct observation

***Adults***	**Direct observation**		**Previous-day recall vs. Direct observation**		
	**N**	**Minutes**	**% Agree (Kappa)**	**Bias (95% CI)**	**ICC (95% CI)**
		**Mean (SD)**			
Total	27	226.9 (94.3)	NA	28.6 (6.9, 50.3)*	0.81 (0.58, 0.91)*
Home	19	108.9 (77.5)	85.2% (0.58)+	2.8 (-8.8, 14.4)	0.96 (0.91, 0.98)*
Work/School	16	185.4 (114.7)	92.6% (0.85)+	14.2 (-4.0, 32.5)	0.93 (0.86, 0.97)*
Community	20	54.4 (46.4)	92.6% (0.81)+	10.9 (-7.4, 29.2)	0.71 (0.47, 0.86)*
Household activity	27	52.8 (49.5)	100% (#)	7.5 (-3.9, 19.0)	0.84 (0.69, 0.93)*
Work	17	160.9 (103.5)	85.2% (0.71)+	9.7 (-13.8, 33.2)	0.88 (0.75, 0.94)*
Education	5	44.5 (40.8)	85.2% (0.42)+	1.3 (-20.4, 23.0)	0.12 (-0.29, 0.48)
Transportation	18	25.7 (15.4)	88.9% (0.74)+	0.0 (-5.9, 5.8)	0.62 (0.32, 0.81)*
Leisure	23	55.6 (42.1)	74.0% (0.32)+	7.1 (-23.7, 38.0)	0.55 (0.23, 0.77)*
***Adolescents***					
Total	26	220.9 (65.8)	NA	24.4 (10.5, 38.2)*	0.80 (0.47, 0.92)*
Home	24	128.2 (79.4)	100% (1.00)+	21.4 (2.8, 40.0)*	0.84 (0.64, 0.93)*
Work/School	5	87.5 (101.8)	80.8% (0.20)+	3.7 (-23.0, 30.4)	0.47 (0.10, 0.72)*
Community	19	117.2 (78.4)	88.5% (0.66)+	5.9 (-25.0, 36.8)	0.73 (0.48, 0.87)*
Household activity	26	36.1 (32.4)	88.5% (#)	14.3 (-2.7, 31.4)	0.46 (0.12, 0.71)*
Work	2	79.7 (106.9)	92.3% (#)	-10.6 (-25.8, 4.5)	NA
Education	5	85.6 (107.2)	80.8% (0.20)+	-4.2 (-17.7, 9.2)	0.78 (0.58, 0.90)*
Transportation	17	22.1 (13.0)	88.5% (0.76)+	1.8 (-4.7, 8.3)	0.67 (0.39, 0.84)*
Leisure	26	147.7 (61.2)	100% (1.00#)	40.4 (-1.1, 82.0)	0.49 (0.13, 0.73)*

Note: N is number of segments in which the location/purpose was observed for at least 1 minute. ICC is intraclass correlation.

*Indicates statistical significance at p < 0.05, #indicates the Kappa was not computable; +indicates agreement is not by chance according to McNemar Test.

## Results

The adolescent participants in the current analysis were similar in age, BMI, sedentary time and sex distribution to the larger sample (Table 1). The adult participants in the current analysis were slightly younger than the larger convenience sample of community-living adults (P < 0.05), but were similar in BMI, sedentary time and sex distribution (Table 1). Participant characteristics and average time in each segment of the day for PDR and direct observation are shown in Table 1.

**Figure 1:**
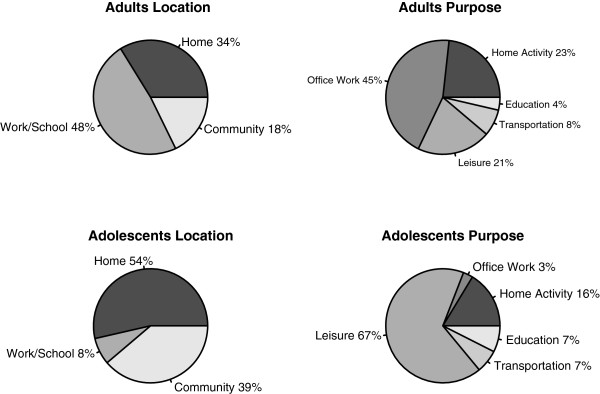
**Description of time in each location and purpose according to direct observation.** Note: Adults N = 27 segments, average observation time was mean (SD) 226.9 (93.4) min per segment, adolescents N = 26 segments, average observation time was 220.9 (65.8) min per segment.

Figure 1 shows the percent of observed time spent in each location and purpose. For adults, there were 27 segments observed and the segments lasted 226.9 min on average. The majority of observed time was spent at work/school (48%), followed by home (34%), and community (18%) locations. For purpose, adults spent the most time doing work (45%), household activities (23%), and leisure activities (21%). Adolescents were observed for 26 segments that averaged 220.9 min. Most of the observed time was spent at home (54%) followed by time in the community (39%), and only 8% was spent at work/school. Adolescents spent most of their time in leisure activities (67%), followed by household activities (16%). Several categories had five or fewer observations. For completeness we report all results in our tables, but given the likely imprecision in these estimates for categories with 5 or fewer observations we do not interpret these results.

### Total time reported, by location and purpose

For adults, the comparisons between direct observation and PDR for each location and purpose are shown Table 2. All estimates of bias and ICC are presented as value (95% CI) unless otherwise indicated. Adults reported 28.6 (6.9 to 50.3) min more of total time on the PDR than direct observation time. For activity location the percent agreement was 85% or greater for each location and Kappa values were moderate to strong, ranging from 0.58 to 0.85. There was a non-significant positive bias for the work/school and community locations and ICCs in each location were significant and relatively high (ICC ≥ 0.71). There was no statistically significant bias between PDR and direct observation for any purpose. Reported classification of activity purpose between the two methods was 74% or greater. Kappa statistics ranged from fair (0.32) for leisure activities to substantial agreement in transportation (0.74). The ICC for purpose among adults was lowest for education (0.12 [-0.29 to 0.48]) and highest for household activities (0.96 [0.91 to 0.98]).

Adolescents reported 24.4 (10.5 to 38.2) min more on the PDR than direct observation (Table 2). For location, the percent agreement was 80% or greater for each location and Kappa values were substantial for the community (0.66), perfect for home (1.0), but only slight for work/school (0.20), where only 5 observations were available for analysis. There was a significant positive bias for the home location of 21.4 (2.8 to 40.0) min, but ICCs in each location were significant and relatively high (ICC ≥ 0.47). For the activity purposes there were no significant biases though the confidence intervals were very wide for leisure activities, which were overestimated by 40.4 (-1.1 to 82) min. Bias was smallest for transportation at 1.8 (-4.7 to 8.3) min. Reported classification of activity purpose revealed percent agreement that was 80% or greater and Kappas that ranged from 0.20 for education to 1.0 for leisure activities. The ICCs were all significant, ranging from 0.46 to 0.78.

### Physically active and sedentary behaviors, by location and purpose

For adults, 59% (133.9 ± 76.0 min) of the observed time was spent sedentary and the remainder was spent in active behaviors (92.9 ± 63.4 min). The bias, percent agreement, Kappa and ICCs comparing direct observation and PDR by active and sedentary behaviors are shown in Table 3. There were no significant differences between PDR and direct observation estimates of active time at home (mean [95% CI]) (-1.6 [-11.5 to 8.3] min) and active time at work/school (5.3 [-12.9 to 23.5] min) and the percent agreement and Kappas were substantial for both locations. For community, the estimates of active time were similar between methods (5.7 [-7.8 to 19.2] min) and the percent agreement (74.1%) and Kappa (0.49) and were moderate, but the ICC was not statistically significant 0.28 (-0.12 to 0.59). For activity purpose, the bias was smallest for leisure time (0.1 [-20.0 to 20.2] min) and highest for work, with PDR estimating 8.3 (-12.8 to 29.4) min higher than direct observation. The percent agreement and Kappa were lowest for leisure activities (59.3% and 0.27) and highest for work (85.2% and 0.71). ICCs were moderate for home activity, work and leisure activities (0.51 to 0.88), but not for transportation (-0.08).

**Table 3:** Comparison of adults time spent in active and sedentary behaviors by location and purpose using previous day recall and direct observation

	**Direct observation**		**Previous-day recall vs. Direct observation**		
	**N**	**Minutes**	**% Agree (Kappa)**	**Bias (95% CI)**	**ICC (95% CI)**
		**Mean (SD)**			
***Active***					
Total	27	92.9 (63.4)	92.6% (0.72)+	9.1 (-13.3, 31.5)	0.78 (0.58, 0.89)*
Home	19	52.3 (55.0)	88.9% (0.72)+	-1.6 (-11.5, 8.3)	0.92 (0.84, 0.96)*
Work/School	16	71.0 (64.3)	88.9% (0.78)+	5.3 (-12.9, 23.5)	0.83 (0.66, 0.92)*
Community	20	18.9 (18.5)	74.1% (0.49)+	5.7 (-7.8, 19.2)	0.28 (-0.12, 0.59)
Household activity	26	34.1 (41.3)	81.5% (0.24)+	5.1 (-3.2, 13.4)	0.88 (0.76, 0.94)*
Work	16	62.6 (60.9)	85.2% (0.71)+	0.6 (-16.6, 17.9)	0.83 (0.65, 0.92)*
Education	3	5.3 (6.2)	85.2% (-0.06)+	9.1 (-10.7, 28.9)	-0.01 (-0.39, 0.37)
Transportation	14	7.7 (8.1)	63.0% (0.27)+	-1.0 (-4.7, 2.6)	-0.08 (-046, 0.31)
Leisure	22	22.6 (20.0)	59.3% (0.27)	6.1 (-8.1, 20.3)	0.51 (0.18, 0.74)*
***Sedentary***					
Total	27	133.9 (76.0)	100% (0.72)+	19.8 (-1.3, 41.0)	0.79 (0.58, 0.90)*
Home	18	59.8 (43.6)	85.2% (0.67)+	5.2 (-3.3, 13.8)	0.90 (0.80, 0.95)*
Work/School	14	130.7 (89.7)	88.9% (0.78)+	9.9 (-9.9, 29.7)	0.87 (0.73, 0.94)*
Community	18	39.5 (44.5)	85.2% (0.67)+	6.1 (-3.8, 16.0)	0.92 (0.84, 0.96)*
Household activity	26	20.7 (19.8)	88.9% (0.36)+	5.5 (-8.5, 19.6)	0.58 (0.26, 0.78)*
Work	16	108.4 (76.5)	81.5% (0.64)+	8.3 (-12.8, 29.4)	0.82 (0.64, 0.91)*
Education	4	51.7 (35.1)	92.6% (0.63)+	-6.0 (-12.4, 0.4)	0.44 (0.10, 0.70)*
Transportation	18	19.7 (11.2)	92.6% (0.83)+	1.1 (-3.0, 5.2)	0.81 (0.62, 0.91)*
Leisure	19	41.2 (38.8)	74.1% (0.47)+	0.1 (-20.0, 20.2)	0.63 (0.33, 0.81)*

Note: N is number of segments in which the location/purpose was observed for at least 1 minute. ICC is intraclass correlation.

*Indicates statistical significance at p < 0.05, #indicates the Kappa was not computable. +indicates agreement is not by chance according to McNemar Test.

For sedentary behaviors by location, there was no significant bias, the Kappas were all substantial (0.67 to 0.78), percent agreement was between 85.2 and 88.9%, and ICCs were excellent, ranging from 0.87 to 0.92. For activity purpose, the bias was smallest for leisure time (0.1 min [-20.0 to 20.2] min) and highest for work, with PDR estimates 8.3 (-12.8 to 29.4) min higher than direct observation. Percent agreement ranged from 74.1% for leisure activities to 92.6% for transportation. The Kappas ranged from 0.36 for home activity (fair) to 0.83 for transportation (strong). The ICCs ranged from 0.58 (0.26 to 0.78) for home activity to 0.82 (0.64 to 0.91) for work (Table 3).

For adolescents, 63% (139.0 ± 63.8 min) of the observed time was spent sedentary and the remainder was spent in active behaviors (81.9 ± 47.6 min) (Table 4). The active time reported from PDR was not significantly different than direct observation for any location. For community, percent agreement was 84.6%, the Kappa was 0.57, and the ICC was 0.61 (0.31 to 0.80). For the home location, the percent agreement was 65.4%, the Kappa was low (0.20), and ICC was 0.62 (0.32 to 0.81). There were only five observations of active behavior in the work/school location. For activity purpose, there was a slight underestimation of time in active transportation by -2.5 (-4.6 to -0.3) min and no other statistically significant bias. Percent agreements ranged from 53.8% for transportation to 84.6% for leisure and the Kappas were slight-to-fair. The ICCs were all statistically significant except for work and educational activities, which were only completed in two and four segments, respectively.

**Table 4:** Comparison of adolescent’s time spent in active and sedentary behaviors by location and purpose using previous day recall and direct observation

***Total***	**Direct observation**		**Previous-day recall vs. direct observation**		
	**N**	**Minutes**	**% Agree (Kappa)**	**Bias (95% CI)**	**ICC (95% CI)**
		**Mean (SD)**			
***Active***					
Total	26	81.9 (47.6)	76.9% (0.54)	-3.6 (-18.5, 11.3)	0.73 (0.48, 0.87)*
Home	24	37.9 (33.8)	65.4% (0.20)	-12.1 (-24.4, 0.1)	0.62 (0.32, 0.81)*
Work/School	*5*	*41.6 (71.3)*	*80.8% (0.20)+*	*-4.0 (-17.7, 9.8)*	*0.10 (-0.31, 0.46)*
Community	19	53.3 (39.5)	84.6% (0.57)+	11.3 (-7.2, 29.7)	0.61 (0.31, 0.80)*
Household activity	25	22.9 (31.5)	65.4% (0.12)	6.3 (-10.1, 22.8)	0.56 (0.23, 0.77)*
Work	2	46.9 (60.6)	92.3% (#)	-6.3 (-15.0, 2.5)	NA
Education	4	31.0 (61.5)	88.5% (0.36)+	-4.2 (-12.4, 3.9)	0.24 (-0.15, 0.57)
Transportation	14	7.3 (5.8)	53.8% (0.12)	-2.5 (-4.6, -0.3)*	0.37 (0.01, 0.65)*
Leisure	26	47.6 (38.1)	84.6% (#)	4.2 (-9.6, 17.9)	0.69 (0.43, 0.85)*
***Sedentary***					
Total	26	139.0 (63.8)	94.9% (0.90)+	28.0 (16.4, 39.6)*	0.83 (0.34, 0.92)*
Home	24	90.4 (70.3)	100% (1.00)+	32.2 (20.4, 44.1)*	0.85 (0.25, 0.95)*
Work/School	3	76.6 (46.2)	80.1% (0.34)+	0.0 (-13.0, 13.0)	0.37 (-0.03, 0.66)*
Community	15	81.0 (57.1)	96.2% (0.92)+	-4.7 (-18.1, 8.7)	0.86 (0.71, 0.93)*
Household activity	25	14.6 (10.3)	84.6% (0.29)+	8.4 (1.5, 15.2)*	0.33 (0.02, 0.62)*
Work	1	65.5 (NA)	96.2% (#)	-4.4 (-10.8, 2.0)	NA
Education	4	76.1 (50.0)	84.6% (0.26)+	-0.6 (-11.3, 10.1)	0.75 (0.52, 0.88)*
Transportation	13	21.0 (14.5)	100% (1.00)+	4.4 (-1.2, 10.1)	0.70 (0.43, 0.85)*
Leisure	26	100.2 (57.4)	96.2% (#)	37.0 (0.2, 73.8)*	0.55 (0.18, 0.78)*

Note: N is number of segments in which the behavior was observed for at least 1 minute. ICC is intraclass correlation *indicates statistical significance at p < 0.05, #indicates the Kappa was not computable; +indicates agreement is not by chance according to McNemar Test.

For sedentary behaviors at home, the bias was significant (32.2 [20.4 to 44.1] min); however percent agreement and Kappa were 100% and 1.0, respectively, and the ICC was excellent 0.85 (0.25 to 0.95). There was no significant bias and both Kappas and ICCs were good to excellent for work/school and community locations. Sedentary leisure activities were overestimated by 37.0 (0.2 to 73.8) min and home activities were overestimated by 8.4 (1.5 to 15.2) min. The bias for the remaining activity purposes ranged from -0.6 to 4.4 min. For sedentary activity purpose, percent agreement ranged from 84.6 to 100%, the Kappas were between fair (0.26) and very strong (1.0), and ICCs ranged from 0.33 to 0.75, which were statistically significant (Table 4).

## Discussion

In this study, adults accurately reported that they spent most of their time at work/school and doing office work, such as filing papers, desk work, and working at the computer. In contrast, adolescents most accurately reported their time out in the community and in leisure pursuits, such as watching television, talking with friends, or playing games. In general, participants were most accurate in classifying the location and purpose of the behaviors in which they spent the most time. Adults tended to report the location and purpose of their behavior more accurately than adolescents. This may be partially due to the more structured nature of adults activities (e.g., time at work) compared to leisure activities that were more commonly reported by adolescents. The accuracy in estimating location and purpose was similar for active and sedentary behaviors, with the exception of transportation where adult participants reported sedentary transport time more accurately than active transport time. Our study suggests that participants report the location of their activities with considerable accuracy and also report useful information about the purpose of behaviors, particularly those in which they spend the most time. To our knowledge, this is the first study to validate estimates of behavioral context for active and sedentary behaviors compared to a criterion measure of direct observation.

There are other methods available to gather location and purpose of physical activities and sedentary behaviors. Some questionnaires include contextual information to improve estimates of duration, intensity and frequency of physical activity or sedentary behavior [27,29,30]. Questionnaires typically ask about time domains of activity (e.g., transportation, household, leisure and occupation) and are often validated compared to an activity monitor. The domain specific constructs have not been routinely validated [31,32], in part because of the absence of strong domain-specific criterion measures. As more research has focused on domain-specific associations, researchers may be interested in validating these specific constructs but it is challenging to do so. Some studies use a behavioral log as a criterion, where people record all activities to validate the questionnaire but this still relies on the participants record rather than an objective measure [29]. The gold-standard is direct observation, which is costly, time-intensive and logistically challenging. Other objective methods (described below) may be able to estimate location and purpose of activity and be used as a criterion measure in future studies.

Activity monitors used in combination with GIS and SenseCam technology objectively assess the context of active and sedentary behaviors. GIS systems have been used to explicitly link location-specific physical activity outcomes with attributes of the built environment (e.g., walkability) [33-35]. While GIS is able to characterize where physical activity or sedentary behavior is taking place, this system cannot directly assess the purpose of the behavior. The SenseCam takes first-person pictures of the environment approximately every 20 seconds [36]. The pictures are manually annotated and classify the activity context into categories based on the Compendium of Physical Activities [12,22,37]. This information is combined with accelerometer data to estimate the purpose of active and sedentary behaviors and identify sedentary behaviors that were misclassified by an accelerometer [12,37]. However, when attempting to classify free-living behavior, only 81% of episodes could be classified [37]. Both ethical (e.g., privacy) [38] and practical barriers (e.g., poor lighting) currently limit widespread application of this technology [11]. Further, both GIS and SenseCam require expensive equipment and time-intensive data processing, thus these methods may be less feasible for use in large-scale epidemiological studies. Ecological momentary assessment uses a cell phone platform to gather information about the context and affect within a naturalistic setting [14,39]. This method provides valuable insight into what the participant is doing at an exact moment in time but it does not gather estimates of total time in either active or sedentary behaviors in a given context, as participants are prompted about their current activity multiple times throughout the day [13,39,40].

Understanding where and why active and sedentary behaviors take place can inform individual-level and environmental-level interventions targeting these behaviors [10,41]. All of the tools described above have both strengths and weakness and the choice of instrument should be driven by the research question of interest. For example, if a researcher is increased in long-term activity (i.e., over the past year) then a questionnaire could be preferable to the PDR [30]. Studies examining trips to a particular green-space or trail may consider GPS. Conversely, if the researcher is interested in the location and purpose of active and sedentary behaviors that are currently performed by the individual, the PDR may be preferred. The short-term recall reduces challenges associated with recalling activity over a long prolonged period and gathers detailed information on behavioral context, posture and activity intensity [17,19]. The current data demonstrate that the PDR is a valid tool for measuring location and purpose of physically active and sedentary behaviors compared to direct observation. Previous reports demonstrate its validity for estimating posture and intensity [15], with correlation coefficients that tended to be higher than those of questionnaires that rely upon long-term recall to estimate time spent in different behaviors [31]. The development of self-administered short-term recalls furthers the potential utility of this approach in large-scale epidemiologic studies [16-18].

Despite efforts to match the direct observation time with specific PDR segments, there was still approximately 9% more time reported in the PDR than for direct observation. Compared to direct observation, adults reported 28.6 (6.9, 50.3) min more and adolescents reported 24.4 (10.5, 38.2) min more on the PDR. This difference may be due to errors recalling when a segment started and stopped. For example, if the observation started at 11:50 AM and the participant reported eating lunch at 12:00 PM, there will be 10 additional minutes in the observation period that were not reported in the PDR. Because the PDR used in this study was not time stamped, it was not possible to distinguish error due to incorrect recall of specific activity versus error due to difficulties remembering when the segment started and stopped. For the majority of the segments this was not a major problem, but there were two segments included in the dataset where participant reported the segment length was >1hour more than the observation time, increasing bias. This error recalling when a segment starts and stops may be an artifact of the current study design as it was necessary for us to perform direct observation during segments of the day due to the infeasibility of observing participants for 24 consecutive hours.

The use of direct observation as a criterion is an important strength of this study. The challenges of implementing criterion measures such as direct observation to assess location and purpose of activity may explain the paucity of data in this area. Furthermore, we matched the direct observation system to the PDR in order to ensure both our criterion measure and the PDR were measuring the same constructs. This study has other important strengths as well. The study participants completed a range of activities and the time distribution of activities was fairly consistent with how Americans typically spend their time outside of work/school activities [21,42]. Inclusion of a range of locations and purpose supports the use of the PDR in future studies where participants may complete a variety of behaviors.

There are limitations of the current study that should be considered. Participants may recall their behavior better because they were being observed, though there is no way to empirically test this. To mitigate this effect, the participants were not told the PDR was going to be conducted the following day. The ICCs between direct observation and PDR active and sedentary time in this study (0.73 to 0.83) were similar to the correlations between activPAL and PDR estimates from previous work (0.75 to 0.81), with the exception of adolescent girls who had lower correlations (0.64 to 0.80) in the previous study [14]. This suggests, at least for active and sedentary time, that the accuracy of the recall was not substantially impacted by the observation session. Another limitation to our study is that the sample size was small and may not generalize to other populations. In particular, the sample was relatively young (range 12-62y) with a mean of 42y for adults. Future studies should assess the validity of the PDR with an older adult population. The sample did include a range of BMI (15.7 to 41.0 kg/m^2^) and a roughly equal distribution of males and females (Table 1). There are challenges for both the observer and the PDR when they complete multiple activities at the same time, which could have introduced error. The observers were instructed to code the purpose for the primary activity. For example, if a participant was eating while watching TV, this would be coded as “self-care”, until the participant stopped eating and then it would be considered leisure time. Similarly, for the recall participants were instructed to code the primary activity. If the participant reported doing two activities with substantially different energy expenditure (e.g., reading and walking on a treadmill) the activity with the higher energy expenditure was recorded.

For some locations and purposes, there were a very small number of participants who completed the activity. The accuracy for these less common physical behaviors could not be addressed. It is possible this reflects the actual portion of time individuals spend in these activities (e.g., adults doing educational activities) [42]. For other activities, such as time at work/school for adolescents, the low number of segments (n = 5) was largely due to safety and logistic concerns of the school district in having an outside observer in the classroom. Time at school makes up a large portion of an adolescent’s day and future studies should attempt to validate measurement tools within a school setting.

## Conclusions

Our study suggests that adults and adolescents accurately report where (location) and why (purpose) they spent the majority of their time using a PDR. A range of methods are available to capture information about the location of human activities, including GPS and photographic monitoring, but few methods other than self-report gather details about the purpose of a given behavior. Our results support the idea that PDRs can provide important contextual details that can be used as stand-alone measures or in combination with objective measures. The ability to determine the location and purpose of active and sedentary behaviors may help inform behavioral theories, health interventions and public policy.

## Competing interests

The authors declare that they have no competing interests.

## Authors’ contributions

Conceived of experiment: SKK KL, PF, CM, data collection and statistical analysis: KL, AH, ER, SKK, CM, wrote the paper: SKK, KL CM, provided edits and comments SKK, AH, ER, KL, CM, JF, PF. All authors read and approved the final manuscript.
